# Epidemiology of Human Respiratory Viruses in Children with Acute Respiratory Tract Infections in Jinan, China

**DOI:** 10.1155/2013/210490

**Published:** 2013-12-02

**Authors:** Yanqin Lu, Shifu Wang, Lehai Zhang, Chao Xu, Cuirong Bian, Zhaoxia Wang, Yanhui Ma, Ke Wang, Lixia Ma, Chen Meng, Caiyun Ni, Jiabei Tong, Gongchao Li, Jinxiang Han

**Affiliations:** ^1^Shandong Medicinal Biotechnology Centre, Key Laboratory for Modern Medicine and Technology of Shandong Province, Key Laboratory for Virology of Shandong Province, Key Laboratory for Rare & Uncommon Diseases of Shandong Province, Key Laboratory for Biotech-Drugs, Ministry of Health, Shandong Academy of Medical Sciences, No. 18877 Jingshi Road, Jinan 250062, China; ^2^School of Medicine and Life Sciences, University of Jinan-Shandong Academy of Medical Sciences, Jinan 250200, China; ^3^Department of Laboratory, Qilu Children's Hospital of Shandong University, Jinan 250022, China; ^4^School of Dental Medicine, Shandong University, Jinan 250012, China; ^5^Institute of Basic Medicine, Shandong Academy of Medical Sciences, Jinan 250062, China; ^6^Respiratory Department, Qilu Children's Hospital of Shandong University, Jinan 250022, China

## Abstract

The viral etiologies of UTRIs and LTRIs in children in Jinan city were investigated between July 2009 and June 2010. Nasal and throat swabs were collected from 397 children with URTIs and bronchoalveolar lavage fluid specimens were collected from 323 children with LRTIs. RT-PCR/PCR was used to examine all samples for IFV, PIV, RSV, RV, hMPV, HBoV, CoV, ADV, RSV, and EV. Viral pathogens were detected in 47.10% of URTI samples and 66.57% samples, and the incidence of viral coinfection was 5.29% and 21.05%, respectively. IFV was the most common virus in URTIs, with a detection rate of 19.40%, followed by PIV (10.83%), RV (10.58%), and EV (6.30%). For LRTIs, PIV and RV were both detected in 27% of samples, followed by RSV (9.91%), HBoV (8.36%), IFV (5.57%), and hMPV (5.57%). RSV and HBoV were more prevalent in the youngest children of no more than six months. Meanwhile, RV, PIV, and RSV were the most frequent viruses combined with bacterial pathogens in LRTIs. In conclusion, the spectrum of respiratory virus infections in URTIs and LRTIs differed in terms of the most common pathogens, seasonal distribution, and coinfection rate.

## 1. Introduction

Acute respiratory tract infection (ARTI) is a persistent and pervasive public health issue and a great burden to both families and the wider society. Acute low respiratory tract infection is a particular problem, being the principal cause of morbidity and mortality in young people worldwide [1–3]. The most common viral causes of ARTI worldwide include respiratory syncytial virus (RSV), parainfluenza viruses (PIVs), influenza viruses (IFVs), enteroviruses (EVs), adenoviruses (ADVs), human rhinoviruses (HRVs), human metapneumovirus (hMPV), and human coronaviruses (HCoVs) 229E, OC43, NL63, and HKU1. There are an increasing number of cases with severe acute respiratory syndrome caused by coronaviruses, including NL63, HKU1 [4–6], and human bocavirus [7], and the WU and KI polyomaviruses have been discovered to cause acute respiratory tract infections [8, 9].

The pattern of respiratory tract infections is variable and is related to factors that include region, season, and year [10, 11]. The virologic epidemiology of ARTIs in children has been investigated in various regions [12–14]. In this study, we aimed to characterize the viral spectrum and pattern of upper and lower ARTIs in children in Shandong province, China.

## 2. Materials and Methods

### 2.1. Ethics Statement

The study followed the Declaration of Helsinki on medical protocol and ethics. The Ethics Committees of both Shandong Medicinal Biotechnology Centre and Qilu Children's Hospital of Shandong University approved the study. Written informed consent was obtained from the next of kin of the participants.

### 2.2. Clinical Specimens

Respiratory secretions were obtained from patients aged less than 14 years old with acute respiratory tract infections at Qilu Children's Hospital of Shandong University, China, between July 2009 and June 2010. Nasal and throat swabs were collected from children with acute upper respiratory tract infection (URTI) at the outpatient department and bronchoalveolar lavage fluid specimens were obtained from inpatients diagnosed with acute lower respiratory tract infection (LRTI). Nasal and throat swabs collected from the same children were pooled into a single tube containing virus transport medium (VTM, Copan, Brescia, Italy). The clinical symptoms of subjects are summarized in Table 1. Patients with URTIs enrolled in the study were selected by physicians according to the presence of one or more respiratory symptoms as described previously [15]. In addition to the symptoms of URTIs, patients with radiological pulmonary abnormalities, such as evidence of pneumonia, bronchopneumonia, increased lung markings, dyspnea, or abnormal pulmonary breath sounds, were diagnosed with a LRTI. For patients with LRTIs, sputum or blood samples were collected and cultured for bacteria.

### 2.3. Nucleic Acid Extraction

Total nucleic acids including DNA and RNA were extracted from 200 *µ*L of each specimen using a QIAamp MinElute Virus Spin Kit (Qiagen, Mississauga, ON, Canada) according to the manufacturer's instructions.

### 2.4. PCR/RT-PCR Screening for Respiratory Viruses

For all collected specimens, PCR or RT-PCRs were performed to detect infection with ADVs, HBoV, HCoV, hMPV, IFVs, RSV, PIV, EV, and HRV. The method used for each virus is described in the following references: PIVs 1–4, EVs, HRVs [16], IFVs A, B, and C, and RSVs A and B [17] were detected by two multiplex nested RT-PCRs; HCoVs [6] and hMPV [18] by two-step RT-PCR; ADVs [19] by single-step PCR; and HBoV by touch-down PCR [20]. RT-PCR was performed using a SuperScript II one-step RT-PCR Platinum Taq kit (Invitrogen, Carlsbad, CA, USA). PCR was performed using ExTaq polymerase (Takara, Otsu, Japan). All products underwent electrophoresis in 2% agarose gel. Typing for IFV, PIV, and RSV was performed according to PCR product size.

## 3. Results

### 3.1. Prevalence of Respiratory Viruses

**Figure 1:**
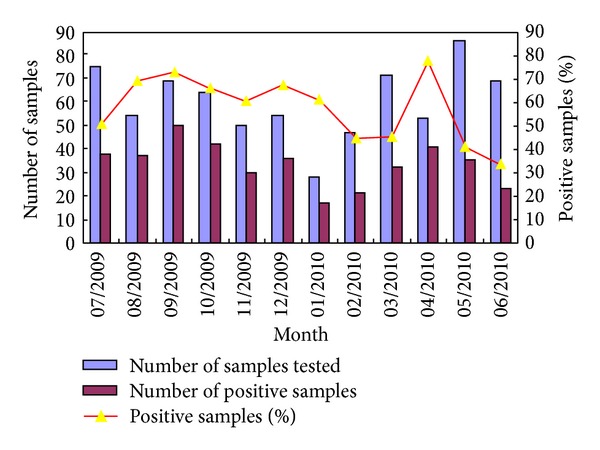
Number of recruited patients and the numbers of positive samples for viral infections.

From July 2009 to June 2010, 397 nasal and throat swabs were collected from outpatients and 323 bronchoalveolar lavage fluid specimens were collected from inpatients. Samples were collected twice weekly (every Tuesday and Friday). In January 2010, seven bronchoalveolar lavage fluid specimens and 21 swabs were collected; the total number was lower than that in the other months (Figure 1). Overall, 402 out of 720 samples (55.83%) were found to be positive for at least one respiratory virus. The virus detection rate for URTIs and LRTIs was 47.10% and 66.57%, respectively. Ninety-five samples (13.19%) contained two or more viruses, and the coinfection rate of more than one virus was 6.05% and 25.98% for URTIs and LRTIs, respectively. The most prevalent viruses detected were RV (19.72%), PIV (17.78%), and IFV (13.9%).

### 3.2. Differences in the Respiratory Viral Spectrum between Upper and Lower ARTIs

The respiratory viruses found in URTIs and LRTIs are shown in Table 2. IFV, PIV, and RV were the most commonly detected viruses in URTIs, at rates of 19.40%, 10.83%, and 10.58%, respectively. RV, PIV, RSV, and HBoV were the principal viruses detected in LRTI samples, at rates of 27.86%, 26.32%, 9.91%, and 8.36%, respectively. The positivity rates for PIV and RV were similar in both URTI and LRTI samples. ADV, CoV, and hMPV had low detection rates in both URTI and LRTI samples. HBoV and RSV were found in 8.36% and 9.91% of LRTIs but had a low incidence (<1%) in URTIs. The predominant subtype of influenza A, respiratory syncytial virus type B, and human parainfluenza virus type 3 accounted for higher percentage of the positive viral detections in both URTIs and LRTIs (Table 2).

### 3.3. Seasonality of Respiratory Virus Infection

**Figure 2:**
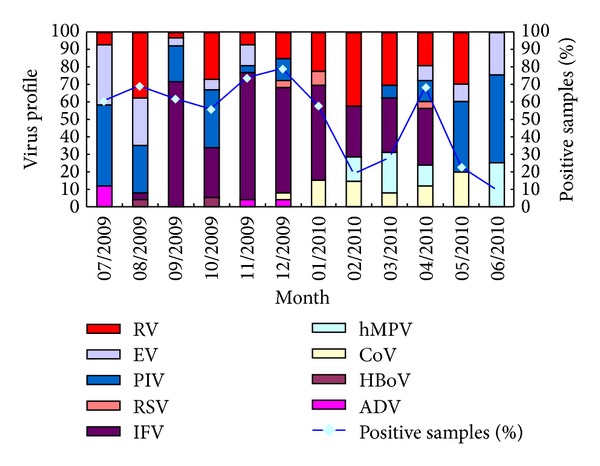
Number of positive results for various viruses in patients with acute upper respiratory tract infections.

**Figure 3:**
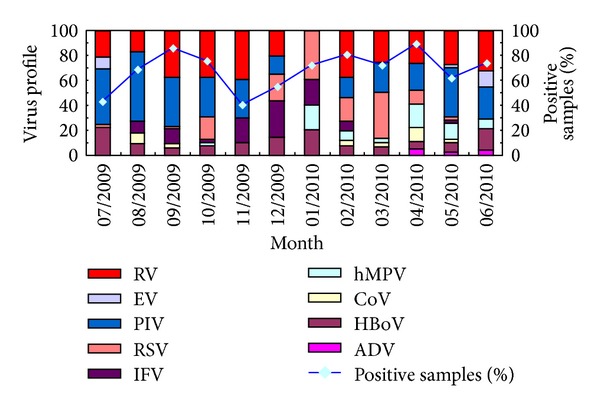
Number of positive results for various viruses in patients with acute lower respiratory tract infections.

The distribution of viruses by month somewhat differed between URTIs and LRTIs (Figures 2 and 3). In URTIs, IFV first appeared in August 2009; its positivity rate was 70.83% in September 2009, which decreased to 27.78% in October and reached a peak of 70.83% in November. A minor peak in IFV (32%) occurred in April. No IFV infection was observed in July 2009 or in May or June 2010. Similar seasonality was observed in LRTIs, with IFV having a high prevalence during autumn and winter.

The trend for PIV differed from that of IFV. In UTRIs, PIV had a high prevalence (40–50%) in July 2009 and in May and June 2010 and a low prevalence (0–12%) from December 2009 to April 2010. Similar seasonality was observed for IFV in LRTIs; its peak occurred in summer and autumn, but its total positivity rate was only 5.64%.

RVs were the third most common virus in URTIs and the most prevalent in LRTIs. They were detected throughout the year, with a low rate in summer in URTIs. No seasonality was observed for RVs in LRTIs.

The prevalence of EV was similar in URTIs and LRTIs. The highest detection rate was in summer; no EV infections were observed in winter or autumn. The positivity rate for EV was 11.57% in URTIs and 2.19% in LRTIs.

RSV was found throughout the year in LRTIs; the highest detection rate (10.97%) was in winter, followed by spring. In URTIs, its detection rate was 1.39%, and it occurred sporadically during the year of the study.

HBoV was also present throughout the year in LRTIs, with two small peaks in winter and spring. In URTIs, its detection rate was 0.92%, and it occurred sporadically throughout the study.

### 3.4. Age Distribution of Respiratory Virus Profiles

**Figure 4:**
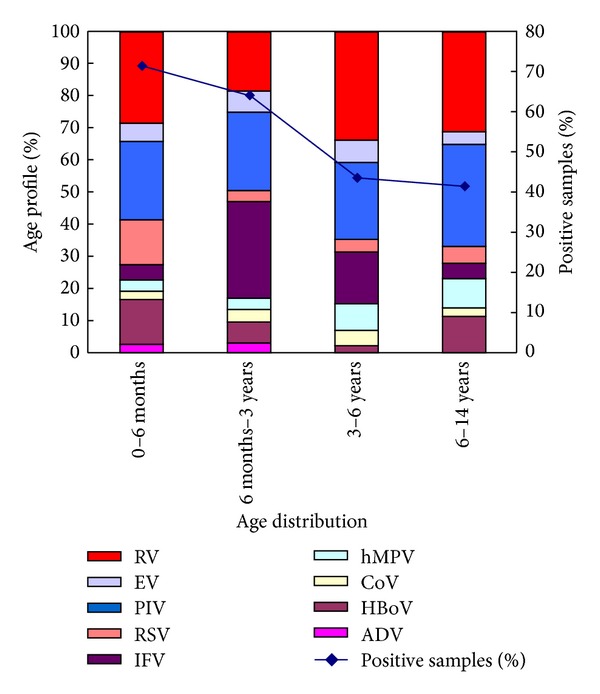
Percentage of samples that was positive for various viruses in different age groups of patients.

The positive detection rates of viruses that corresponded to different age groups are shown in Figure 4. RV was the most prevalent virus, detected in 31.82% of children aged less than 6 months, 18.89% in children aged 3–6 years, and 18.66% in children aged 6–14 years. Among the younger age group (6 months–3 years), IFV had the highest prevalence (23.65%). PIV was the second most prevalent virus in all age groups and the joint leading viral cause in children older than 6 years. RSV and HBoV were the most significant agents in the youngest age groups compared to children older than 6 months, at an equal rate of 15.45%. The least common viruses in all age groups were ADV, CoV, and hMPV.

### 3.5. Codetection of Respiratory Viruses

The coinfection percentage of multiple respiratory viruses differed between URTIs and LRTIs. Ninety-nine patients were found to be coinfected in total; 21 (5.29%) had URTIs and 68 (21.05%) had LRTIs. The pattern of coinfection was complex; 17 and 25 different combinations were found in URTIs and LRTIs, respectively. In URTIs, coinfections were found in every month. PIV, detected in 11 cases, was the most common virus in URTI coinfections and was most often found with RV. PIV was also the predominant virus in LRTI coinfections (40 cases). PIV and RV dual infections occurred in September and October 2009. Fourteen cases of triple infection were observed, with the rate of 4.33% (Table 3).

In IRTIs, severe clinical phenotypes, severe pneumonia (11.76%), and heart failure (10.29%) were more prevalent in coinfection patients than patients with monoinfections or no infections. Compared with mono- and noninfection, a lower percentage of positive viral tests (17.65%) were observed in patients with coinfection and pulmonary atelectasis (Table 4).

### 3.6. Viral-Bacterial Coinfections

In all 323 patients with LRTIs, a total of 93 and 48 specimens were positive for pathogenic bacteria and copathogenic bacteria, respectively. *Streptococcus mitis*, *Streptococcus viridans,* and *Klebsiella pneumonia* were the prevalent bacteria with positive rates of 31.91%, 21.99%, and 19.15%, respectively (Table 5). Group D streptococci were the most likely pathogens to be coinfecting, with the positive rate of *S. mitis* of 12.06%. The percentage of viral-bacterial confections was 27.24% and most often viral copathogen was RV, PIV, and RSV. RV was not found to coinfect with group streptococci. PIV was also absent from infections with *S. mitis* and group D streptococci. Contrary to PIV, HBoV was the most frequent viral pathogen to coinfect with *S. mitis* and group D streptococci.

## 4. Discussion

In this study, we investigated the viral etiology of URTIs and LRTIs in 720 children, both outpatients and inpatients, in Jinan city, China, from July 2009 to June 2010. The virus positive rate was approximately 20% higher in LRTIs than in URTIs. This may have been due to lower viral loads in nasal and throat swabs compared with bronchoalveolar lavage fluids [21].

The three principal pathogens in children with ARTIs were RV, IFV and PIV. The epidemiology differed between URTIs and LRTIs. IFV, was the most commonly detected virus in URTIs but was observed at only a low rate in LRTI samples. This is entirely different from the situation in adults. Ren et al. reported that IFV plays a major role in both URTIs and LRTIs in Chinese adults. Also, the detection rate for IFV (19.70%) in adults with URTIs is in accordance with our data from children [22]. Given that the same method of detection was used, these data may indicate that the IFV infection rate is similar in children and adults. The seasonality of IFV in children was also similar to that found in adults. IFV infection was observed most commonly during autumn and winter [22], with no infections in May, July, or June, and only one case was found in August. Considering the fact that the study was performed during the global pandemic H1N1 influence A [23], the peaks of IFV infection in our study, especially for URTIs, could be explained in part by the unusual influenza epidemiology this year, although the children recruited in our study were not attending the specific H1N1 clinic provided by the hospital.

RV and PIV were the predominant viruses found in LRTIs, with a similar detection rate that was as high as 27%. These two viruses were also the most common in the URTI spectrum, with detection rates of approximately 11%. RSV was the next most commonly detected virus after RV and PIV, with a positivity rate of 9.91%.

There are many reports regarding the etiology of LRTIs in children in which the viral spectrum and positivity rate vary between regions and over time. RSV has been reported to be the principal pathogen in LRTIs in many studies, with rates that range from 28.5% to 64.7% [24–26]. In Beijing city, RSV was reported to be the predominant virus in LRTIs in children, with a positive rate of 27%; PIV, MPV, and HCoV were not observed between 2004 and 2006 [27]. Between March 2007 and January 2008, RSV, RV, and PIV were reported to be the three principal viral causes of LRTIs, with detection rates of 48.3%, 27.1%, and 13.3%, respectively [28]. Also in Beijing city, during the period from March 2007 to March 2010, positivity rates for RSV, HRV, and PIV were 50.9%, 36.2%, and 12.0%, respectively, in children aged below 1 year [29]. A report from Changsha city also showed that RSV was prevalent in late autumn and winter, but not in spring or summer [30]. These conflicting results could be explained by regional or environmental variability.

The newly identified parvovirus HBoV has been documented in children with acute respiratory tract infections worldwide, although positivity rates vary between reports. Detection rates of approximately 8% have been recorded in children with LRTIs in both Hunan province and Beijing city [30, 31], and our results were in accordance with these reports. HBoV infection was predominantly detected in children with LRTIs, which conflicts with data reported from Hong Kong, where HBoV was common in both upper and lower ARTIs in children [32].

RV and PIV were found to be prevalent in almost all age groups, while ADV, CoV, and hMPV were the least common viruses among children in all age groups in our study. RSV and HBoV were found to be more prevalent in children aged less than 6 months. Incidence of RSV in children, especially young hospitalized children with LRTIs, was high [33, 34].

PIV coinfection was prevalent in both URTIs and LRTIs in our study, and PIV-RV coinfection was common in LRTIs. Mixed respiratory virus infections are often seen in hospitalized children [35]. In children with URTIs, the incidence of coinfection with IFV has been reported to be as high as 27.6% [28]. In our study, however, IFV coinfections were rare, and the reasons for this need to be explored. The combinations of respiratory virus coinfection are complex. RSV-hMPV and RSV-RV coinfection have been reported with a high incidence in children [35, 36]. Our study found a high prevalence of PIV-RV coinfection, which was reasonable given the high positivity rates for these two viruses. The correlation between coinfection and disease severity is controversial. Children with hMPV-RSV coinfection have more severe symptoms than those with a single infection [37, 38], but a negative correlation between hMPV-RSV coinfection and disease severity has also been reported [39]. In our study, mixed infections were prone to be found in patients with severe clinical symptoms.

There has been no comprehensive study of concurrent bacterial and viral respiratory tract infection diseases. *S. pneumoniae *has been reported to be the most common pathogen in adult patients affected by community-acquired pneumonia (CAP), with mixed IFVA infection [40, 41]. The presence of HBoV with *H. influenzae* concurrent infections increases the risk of acute otitis media in children with URTIs [42]. In our study, a high frequency of HBoV was observed in combination with either *S. mitis* or group D streptococci. The concurrent prevalence of viral-bacterial infections was also evident in other viruses, such as RV, PIV, and RSV.

## 5. Conclusions

In summary, we compared the spectrum, seasonality, age distribution, and coinfection of respiratory virus infections in children with URTIs and LRTIs. Our data show that the viral epidemiology differed between URTIs and LRTIs. It is necessary to monitor respiratory viruses over long periods of time to determine their epidemiology.

## Figures and Tables

**Table:** (a)

Age	3.97 years (1 month–14 years)
Gender (M/F)	245/152
Body temperature (median; range)	38.6 (36–40.2)
patients with clinical symptoms	
Cough	321
Nasal discharge	255
Fever	241
Sore throat	23
Sneeze	11
Expectoration	7
Nausea	3
Headache	2
Diarrhea	2
Tears	2
Muscle aches	2

**Table:** (b)

Age (median; range)	3.07 years (0–13 years)
Gender (M/F)	200/123
Body temperature (median; range)	38.35 (35.5–41.2)
patients with clinical symptoms	
Cough	272
Fever	140
Asthma	61
Expectoration	10
laryngeal stridor	9
Vomiting	7
Diarrhea	5
Trembling	4
Chills	4
Headache	3
Gasping	3
Abdominal distension	3
Convulsions	3
Nasal discharge	2
Chest pain	2

**Table 2:** Positive rate of coinfection with respiratory viruses versus mono-infection in patients with URTIs and LRTIs.

	URTIs (number/percentage)			LRTIs (number/percentage)		
	All	Mono-infection	Co-infection	All	Mono-infection	Co-infection
ADV	5/1.26	2/0.50	3/0.76	5/1.55	1/0.31	4/1.24
HBoV	2/0.50	0	2/0.50	27/8.36	12/3.72	15/4.64
CoV	8/2.02	5/1.26	3/0.76	10/3.1	1/0.31	9/2.79
hMPV	8/2.02	5/1.26	3/0.76	18/5.57	11/3.41	7/2.17
IFV	77/19.40	72/18.14	5/1.26	18/5.57	9/2.79	9/2.79
IFVA	58/14.61	54/13.60	4/1.01	13/4.02	8/2.48	5/1.55
IFVB	14/3.53	13/3.27	1/0.50	5/1.55	1/0.31	4/1.24
IFVC	5/1.26	5/1.26	0	0	0	0
RSV	3/0.76	1/0.25	2/0.50	32/9.91	13/4.02	19/5.88
RSVA	1/0.25	0	1/0.25	4/1.24	3/0.93	1/0.31
RSVB	2/0.50	1/0.25	1/0.25	28/8.67	10/3.10	18/5.57
PIV	43/10.83	32/8.06	11/2.77	85/26.32	44/13.62	41/12.69
PIV1	13/3.27	10/2.51	3/0.76	12/3.72	4/1.24	8/2.48
PIV2	1/0.25	1/0.25	0	0	0	0
PIV3	24/6.05	16/4.03	8/2.02	67/20.74	37/11.46	30/9.29
PIV4	5/1.26	5/1.26	0	6/1.86	3/0.93	3/0.93
EV	25/6.30	19/4.79	6/1.51	4/1.24	3/0.93	1/0.31
RV	42/10.58	33/8.31	9/2.27	90/27.86	43/13.32	47/14.55

**Table 3:** Viral co-infection from both URTIs and LRTIs.

URTIs types	Number	IRTIs types	Number
PIV + EV	2	PIV + EV	1
HBoV + IFV	1	HBoV + IFV	2
RV + hMPV	1	RV + hMPV	1
PIV + RV	4	PIV + RV	16
PIV + ADV	1	PIV + ADV	2
PIV + hMPV	1	PIV + hMPV	2
PIV + IFV	1	PIV + IFV	3
RV + IFV	1	RV + IFV	3
RV + RSV	1	RV + RSV	8
CoV + ADV	1	HBoV + CoV	1
CoV + IFV	1	HBoV + RSV	4
CoV + RSV	1	HMPV + CoV	1
EV + HBoV	1	PIV + CoV	2
EV + RV	1	PIV + RV + CoV	2
HMPV + IFV	1	PIV + RV + hMPV	2
PIV + EV + RV	1	PIV + RV + RSV	5
PIV + EV + ADV	1	PIV + RSV	1
Total	**21**	PIV + HBoV	1
		PIV + HBoV + ADV	1
		PIV + RV + HBoV	2
		PIV + RV + hMPV + RSV	1
		RV + CoV	2
		RV + HBoV	3
		RV + HBoV + IFV	1
		RV + CoV + ADV	1
		Total	**68**

**Table 4:** Comparison between viral infection and clinical severity of LRTIs.

	Number/percentage		
	Mono-infection	Co-infection	No infection
Pneumonia	49/33.33	23/33.82	33/30.84
Pulmonary atelectasis and pneumonia	47/31.97	12/17.65	37/34.58
Capillary bronchitis	2/1.36	1/1.47	0
Bronchopneumonia	26/17.69	13/19.11	18/16.82
Severe pneumonia	7/4.76	8/11.76	3/2.80
Heart failure	5/3.40	7/10.29	2/1.87
Pleural effusion	14/9.52	6/8.82	10/9.35

**Table 5:** The number of patients with a combination of viral and bacterial pathogens in patients with LRTIs.

	Total	*K. pneumonia *	*S. viridans *	Group D streptococci	*S. mitis *
ADV	3	2	1	0	0
HBoV	13	3	1	7	7
CoV	5	0	3	2	2
hMPV	7	3	2	1	1
IFV	10	1	1	4	4
RSV	18	1	2	2	8
PIV	38	14	11	0	0
EV	2	1	1	1	1
RV	42	7	11	0	13
